# Low-intensity mind–body exercise for mental health in ageing societies: Tai Chi as a scalable intervention across depression, anxiety, insomnia, loneliness, and cognitive decline

**DOI:** 10.3389/fpsyg.2026.1917045

**Published:** 2026-10-16

**Authors:** Xiaoqi Zhang, Yaya Shi

**Affiliations:** 1 School of Physical Education and Health Sciences, Guangxi Science & Technology Normal University, Laibin, China; 2 School of Physical Education, Yan’an University, Yan'an, China

**Keywords:** community health, exercise therapy, geriatric psychiatry, meditative movement, mild cognitive impairment, non-pharmacological intervention, sleep quality, social participation

## Abstract

Population ageing is increasing the number of older adults who experience overlapping depressive symptoms, anxiety, insomnia, loneliness, and cognitive vulnerability, often alongside multimorbidity, pain, frailty, and functional limitation. This structured narrative review evaluates Tai Chi as a scalable, low-intensity mind–body exercise for these interconnected problems. We synthesised 171 publications identified through PubMed and citation chaining, including Tai Chi-specific clinical studies, broader mind–body evidence, guidelines, and implementation literature. Evidence is most consistent for reducing depressive symptoms, improving sleep, and improving balance, with possible benefits for selected cognitive outcomes. Evidence is weaker for diagnosed depressive or anxiety disorders, loneliness as a primary outcome, long-term cognitive trajectories, and dementia incidence. Tai Chi should therefore be positioned as an adjunct to established care, not as a replacement for pharmacotherapy, psychotherapy, cognitive behavioral therapy for insomnia (CBT-I), or multidomain dementia-risk reduction. Proposed pathways include autonomic regulation, sleep stabilisation, behavioral activation, balance confidence, cognitive–motor integration, and group participation; however, most mechanism evidence is indirect or exploratory. Translation requires clearly specified protocols, active comparators, blinded outcome assessments where feasible, long-term follow-up, systematic adverse-event reporting, cost-effectiveness analyses, and equity-focused implementation. Tai Chi is the most credible as an adaptable component of stepped or integrated care for older adults, provided that programmes are standardised, safely delivered, and evaluated against clinically significant outcomes.

## 1 Introduction

Population ageing is reshaping global mental-health needs. Mental disorders remain major contributors to years lived with disability, and their burden has not materially declined over recent decades (GBD 2019 Mental Disorders Collaborators, 2022). In later life, depression, anxiety, sleep disturbance, loneliness, frailty, multimorbidity, pain, functional decline, and cognitive vulnerability commonly co-occur. This clustering challenges single-disease care and creates a need for interventions that can address mental, behavioral, social, and functional pathways together (Livingston et al., 2024).

These problems are interconnected rather than independent. Prospective evidence associates insomnia with subsequent depression (Li et al., 2016), while longitudinal studies link sleep disturbance to a higher risk of dementia (Shi et al., 2018). Social isolation and loneliness are associated with mortality and poorer cognitive health (Penninkilampi et al., 2018; Holt-Lunstad et al., 2015). The 2024 Lancet Commission also identifies depression, physical inactivity, and social isolation among modifiable dementia-risk factors, supporting an integrated—but not necessarily causal—prevention framework (Livingston et al., 2024).

Current treatments are essential but do not reach every older adult. Pharmacotherapy can be complicated by multimorbidity, polypharmacy, adverse effects, falls risk, and cognitive vulnerability. Evidence-based psychological treatments, including cognitive behavioral therapy and cognitive behavioral therapy for insomnia (CBT-I), are constrained by workforce, cost, geographical access, and engagement barriers (Edinger et al., 2021). Exercise is supported for depression, and benefits may occur below conventional activity targets (Noetel et al., 2024; Pearce et al., 2022); however, frailty, pain, fear of falling, low motivation, and cognitive impairment can make standard aerobic or resistance programmes difficult to start or sustain.

This gap provides a rationale for low-intensity mind–body exercise, which integrates gentle movement with attention, breathing, balance, body awareness, skill learning, and often group interaction (Larkey et al., 2009; Jahnke et al., 2010). Tai Chi is a useful model because it is low impact, requires little equipment, can be delivered in community or institutional settings, and can be adapted to varied physical and cognitive capacities (Wayne and Kaptchuk, 2008a; Lan et al., 2013). It should be evaluated as a multicomponent behavioral intervention—not as a stand-alone or curative alternative to evidence-based mental-health care (Wayne and Kaptchuk, 2008b).

The evidence is encouraging but uneven. Systematic reviews and network meta-analyses suggest that Tai Chi and related mind–body exercises may reduce depressive and anxiety symptoms in older adults (Kuang et al., 2024; Dong et al., 2024). In chronic insomnia, Tai Chi was inferior to CBT-I immediately after treatment but non-inferior at longer-term follow-up; non-inferiority does not establish equivalence or justify replacing first-line CBT-I (Siu et al., 2025). Meta-analyses report possible cognitive benefits in mild cognitive impairment (MCI), but dementia prevention has not been established (Chen et al., 2024; Shao et al., 2024). A systematic review of exercise in older women also identified Tai Chi among modalities associated with better sleep quality, although its 13 trials varied in exercise type, intensity, and outcome assessment (Khaleghi and Ahmadi, 2024). Across domains, heterogeneity in styles, doses, instructor preparation, comparators, outcomes, follow-ups, and harm reporting limits certainty.

This review therefore considers Tai Chi as a scalable intervention for overlapping late-life vulnerabilities rather than as a treatment for one disease. Its proposed pathways include autonomic regulation, sleep stabilisation, behavioral activation, balance confidence, cognitive–motor integration, and social participation. These pathways are hypotheses to be tested, not established mediators (Livingston et al., 2024; Santini et al., 2020).

We examine depression, anxiety, insomnia, loneliness, and cognitive decline, together with dose, safety, delivery, equity, and implementation. Three evidence layers are kept distinct throughout: direct evidence from Tai Chi or Tai Ji Quan studies; related evidence from Qigong, mindfulness, slow breathing, or broader mind–body exercise; and contextual evidence from exercise psychiatry, social prescribing, falls prevention, and dementia-risk reduction. The aim is to define where Tai Chi may complement established care and what methodological standards are required for credible trials.

## 2 Methods

This structured narrative review and critical synthesis evaluated Tai Chi as a scalable, low-intensity mind–body exercise for five interconnected domains: depression, anxiety, insomnia and sleep disturbance, loneliness and social isolation, and cognitive decline. It also examined proposed mechanisms, dose and fidelity, delivery models, safety, accessibility, health equity, and priorities for implementation research.

### 2.1 Information sources and reproducible search strategy

Medical Literature Analysis and Retrieval System Online (MEDLINE) was searched through PubMed from database inception to 30 June 2026. PubMed was selected as the sole bibliographic database because this clinically oriented narrative review prioritised peer-reviewed biomedical, geriatric, psychiatric, sleep, rehabilitation, and health service literature and benefited from stable Medical Subject Headings (MeSH) indexing. This choice is also a limitation: Sport-science, psychology, regional, Chinese-language, non-indexed, and grey literature may have been missed. Backward citation chaining of key reviews, trials, guidelines, Commission reports, and methodological papers was used to identify additional sources. Searches combined intervention, older-population, outcome, mechanism, and implementation concepts; the exact date-limited PubMed syntax is reproduced in Appendix A.

### 2.2 Eligibility criteria

Eligible peer-reviewed human evidence included Tai Chi, Tai Ji Quan, Tai Chi Chuan, Tai Chi Chih, or a clearly specified Tai Chi-derived protocol in older or ageing populations. Direct clinical evidence was prioritised. Studies of Qigong, meditative movement, mindfulness, slow breathing, or other low-intensity mind–body practices were retained only when they informed a mechanism, comparator, or implementation question and were identified as indirect evidence. Eligible designs included systematic reviews, meta-analyses, randomised trials, pragmatic trials, cohort studies, clinical guidelines, consensus statements, and major methodological or public health reports.

Clinical outcomes included depressive and anxiety symptoms or disorders, insomnia and sleep quality, loneliness and social isolation, cognition and MCI, function, balance, falls, quality of life, and related patient-centred outcomes. Mechanism evidence concerned autonomic regulation, heart rate variability, stress physiology, sleep and circadian pathways, inflammation, neuroplasticity, cognitive–motor integration, self-efficacy, behavioral activation, and social participation. Implementation evidence covered dose, adherence, fidelity, community and primary-care delivery, social prescribing, residential care, rehabilitation, digital delivery, safety, accessibility, equity, and economic evaluation.

We excluded non-human research, studies confined to athletic performance or martial arts competition, acute physiological studies without clinical or mechanistic relevance, and papers in which Tai Chi or related practice was mentioned only incidentally. Because this was a narrative review, no risk-of-bias threshold was used as an exclusion criterion, and no pooled effect estimate was calculated.

### 2.3 Evidence selection and prioritisation

Records were screened in two stages: Titles and abstracts were assessed for topical relevance, followed by full-text assessment against the eligibility criteria. One author performed both screening stages, and the second author verified all inclusion decisions against the eligibility criteria; disagreements were resolved by discussion. Screening was not performed independently in duplicate. Exact duplicates were removed using title, author, year, and digital object identifier. Clinical evidence was prioritised by design strength, comparator credibility, population relevance, outcome validity, and follow-up. The final synthesis comprised 171 unique publications, including direct Tai Chi/Tai Ji evidence and explicitly labelled related or contextual sources. Table 1 summarises 10 key trials. Because the search was iterative and designed for a narrative synthesis rather than a preregistered systematic review, retrieval and exclusion counts were not retained, and a Preferred Reporting Items for Systematic Reviews and Meta-Analyses (PRISMA) flow diagram is not presented. This limits reproducibility beyond the complete search strings and final reference set reported here.

**Table 1:** Characteristics, findings, and limitations of key Tai Chi and related trials relevant to late-life mental health, sleep, function, and cognition.

Study and population	Tai Chi protocol	Comparator	Outcomes and follow-up	Main finding	Major limitations
Lavretsky et al. (2011)	Tai Chi Chih; 2 h/week for 10 weeks, added to escitalopram	Health education and escitalopram	Depression, remission, physical function, cognition, and CRP; assessment at week 14	Compared with health education, Tai Chi Chih produced greater depressive-symptom reduction and higher remission, with gains in physical function and selected cognitive and inflammatory outcomes	Small, single-site adjunct trial; partial responders only; no Tai Chi-only arm
73 adults ≥60 years with major depression and partial response to escitalopram					
Lavretsky et al. (2022)	Tai Chi Chih for 3 months, adjunctive to stable antidepressant treatment	Health education and wellness, adjunctive to the same antidepressant treatment	Mood, cognition, health; 3 and 6 months	Both groups improved in depression; Tai Chi Chih was superior only for general health, not mood or cognition	Attrition (125 completed month 3; 117 month 6); active adjuncts in both groups
178 older adults with major depression receiving stable antidepressants					
Liu et al. (2018)	24 weeks; 60 min, 3 times/week	Usual lifestyle	Depression and HRV; baseline and 24 weeks	Depressive symptoms and selected HRV indices improved; the proposed autonomic interpretation remained exploratory	Small sample; passive comparator; no formal mediation analysis
60 older adults with GDS score ≥10					
Siu et al. (2021)	Yang-style 24-form Tai Chi; 60 min, 3 times/week for 12 weeks	Conventional exercise and no intervention	Actigraphy and subjective sleep; post-treatment and 24 months	Tai Chi and conventional exercise improved objective sleep versus no intervention, with benefits maintained at 24 months	Objective effects were generally similar to conventional exercise; absolute changes were modest; participants were unblinded
320 adults ≥60 years with chronic insomnia					
Siu et al. (2025)	24 one-hour sessions over 3 months	CBT-I	Insomnia Severity Index and secondary sleep outcomes at months 3 and 15	Tai Chi was inferior to CBT-I at month 3 and met the prespecified non-inferiority criterion at month 15	Single-site trial; non-inferiority is not equivalence; does not displace first-line CBT-I
200 adults ≥50 years with chronic insomnia					
Chan et al. (2017)	Peer-assisted Tai Chi Qigong; 60 min twice/week for 3 months plus volunteer contact	Standard care/home visits	Loneliness and social support; 3 and 6 months	Greater improvement in loneliness and satisfaction with social support than standard care, evident in the longitudinal analysis	Pilot sample; hybrid Qigong and volunteer intervention; Tai Chi-specific effects cannot be isolated
48 socially isolated ‘hidden elders’ ≥ 60 years					
Taylor-Piliae et al. (2010)	Six-month class-plus-home Tai Chi programme	Western exercise and attention control	Physical and cognitive outcomes; 6 and 12 months	Improved balance and one cognitive measure; selected effects maintained	Small groups; healthy sample; cognition was secondary; limited clinical generalisability
132 healthy community older adults; mean age 69 years					
Li et al. (2018)	Therapeutic Tai Ji Quan; 60 min twice/week for 24 weeks	Multimodal exercise and stretching	Falls over 6 months	Fall incidence was lower than with multimodal exercise (IRR 0.69) and stretching (IRR 0.42) over 6 months	Falls—not mental health—was the primary endpoint; predominantly White sample
670 adults ≥70 years at high risk of falls					
Li et al. (2022)	Online cognitively enhanced or standard Tai Ji Quan; 60 min twice/week for 16 weeks	Online stretching	Feasibility, retention, safety, exploratory cognition; 16 weeks	Online delivery showed acceptable feasibility, 76% mean attendance, 94% retention, and no major intervention-related moderate or serious events; cognitive outcomes were exploratory	Feasibility study with no inferential efficacy analyses; all participants had internet access
69 community adults with MCI; mean age 74.6 years					
Li et al. (2023)	Online cognitively enhanced or standard Tai Ji Quan; 60 min twice/week for 24 weeks	Online stretching	MoCA and dual-task walking; 16, 24, and 48 weeks	Cognitively enhanced Tai Ji Quan outperformed both standard Tai Ji Quan and stretching for MoCA and dual-task walking at 24 weeks; effects persisted at 48 weeks	No non-exercise control; predominantly high-functioning, educated, White sample; no dementia endpoint
318 adults with MCI or memory concerns					

The table prioritises illustrative trials rather than providing a complete risk-of-bias assessment. CBT-I, cognitive behavioral therapy for insomnia; CRP, C-reactive protein; GDS, Geriatric Depression Scale; HRV, heart rate variability; MCI, mild cognitive impairment; MoCA, Montreal Cognitive Assessment; RCT, randomised controlled trial. Tai Chi Qigong is identified as a hybrid intervention and should not be interpreted as Tai Chi-specific evidence. All entries were checked against the cited primary reports, and comparative claims are stated at the reported assessment points.

### 2.4 Data extraction and synthesis

For each clinical source, one author extracted the population, intervention forms, doses, comparators, settings, outcomes, follow-ups, main findings, safety information, and principal limitations; the second author verified the extracted fields against the source reports. Extraction was not performed independently in duplicate, and disagreements were resolved by discussion. Where reported, we also recorded instructor preparation, tailoring, home practice, adherence, fidelity, and resource use. The findings were synthesised across the five outcome domains and then integrated with mechanism and implementation evidence. Direct Tai Chi findings were not treated as interchangeable with evidence from related practices, and proposed pathways were not described as established mediation unless a study formally tested mediation.

### 2.5 Assessment of evidence strength

No formal risk-of-bias instrument or Grading of Recommendations Assessment, Development and Evaluation (GRADE) rating was applied. Evidence strength was judged qualitatively from design, sample size, comparator, population specificity, measurement validity, consistency, follow-up, and direct relevance to older adults. Greater weight was given to randomised trials, active comparators, objective outcomes or validated outcome measures, and longer follow-ups. Evidence was down-weighted when trials were small, used passive controls, combined several interventions, relied only on self-report, inadequately described the protocol, or failed to report harms. Based on this, evidence is comparatively stronger for depressive symptoms, sleep disturbance, balance, and selected cognitive measures and weaker for diagnosed psychiatric disorders, loneliness as a primary outcome, long-term cognitive trajectories, and dementia incidence.

## 3 Findings

### 3.1 The mental-health challenge in ageing societies

#### 3.1.1 Ageing, multimorbidity, and overlapping mental health burdens

Population ageing increases the time people live with chronic disease, functional limitation, cognitive vulnerability, and social disadvantage. Healthy ageing is therefore defined less by the absence of disease than by functional ability, intrinsic capacity, and participation (Beard et al., 2016). This perspective is directly relevant to mental health because late-life depression, anxiety, sleep disturbance, loneliness, and cognitive decline commonly intersect with pain, disability, dependence, and non-communicable disease (Prince et al., 2015).

Multimorbidity is key to this challenge. Scottish primary-care data showed that physical and mental disorders frequently co-occur and that multimorbidity appears 10–15 years earlier in the most disadvantaged areas (Barnett et al., 2012). Reviews likewise found that the majority of adults older than 65 years live with at least two chronic conditions, although prevalence estimates vary by definition and setting (Salive, 2013; Marengoni et al., 2011). Single-disease services consequently fit many older adults poorly.

Pain, fatigue, sleep problems, reduced autonomy, and functional impairment can contribute to depressive and anxiety symptoms (Zhang et al., 2022; Moussavi et al., 2007); in turn, mental disorders can undermine self-management, physical activity, and function (Fisher et al., 2021; da Rosa et al., 2023). Evaluations should therefore include sleep, function, social participation, physical confidence, and sustained behavior change as well as symptom scores.

#### 3.1.2 Depression, anxiety, insomnia, loneliness, and cognitive decline as interconnected syndromes

Insomnia illustrates the overlap among outcomes. Prospective meta-analyses associate insomnia with later depression (Li et al., 2016; Baglioni et al., 2011), and longitudinal evidence links sleep disturbance to a higher risk of dementia (Shi et al., 2018). These associations make sleep a plausible shared target, but they do not establish that improving sleep through Tai Chi will prevent depression or dementia.

Social disconnection is another shared risk marker. Among older adults, objective disconnection and perceived isolation are associated with depressive and anxiety symptoms (Santini et al., 2020). United States longitudinal data link loneliness and depression to cognitive decline (Donovan et al., 2017), while meta-analytic evidence associates social engagement and loneliness with dementia risk (Penninkilampi et al., 2018). Directionality remains unknown.

Depression is also associated with subsequent dementia, but causal interpretation is difficult. Reviews identify possible causal effects, shared risks, prodromal symptoms, and reverse causation (Byers and Yaffe, 2011; Diniz et al., 2013). Timing and trajectory may modify the association (Lee et al., 2021), and a large Danish cohort found associations across early-, mid-, and late-life depression (Elser et al., 2023). The evidence supports attention to shared modifiable pathways, not a claim that treating depression with Tai Chi prevents dementia.

#### 3.1.3 Limitations of current pharmacological, psychological, and exercise-based care models

Pharmacological treatment remains essential for clinically significant depression, anxiety, insomnia, psychosis, and behavioral symptoms, but later-life prescribing is complicated by altered pharmacokinetics and pharmacodynamics, polypharmacy, drug interactions, falls, cognitive vulnerability, and adverse effects (Mangoni and Jackson, 2004; Hilmer et al., 2007; Maher et al., 2014; American Geriatrics Society Beers Criteria Update Expert Panel, 2023; Seppala et al., 2018). The American Geriatrics Society Beers Criteria identify several commonly used psychotropics as potentially inappropriate in specified circumstances (American Geriatrics Society Beers Criteria Update Expert Panel, 2023). Observational associations between antidepressants and falls or fractures require caution because of confounding by indication (Coupland et al., 2011), while antipsychotic use in dementia requires explicit risk–benefit assessments (Maust et al., 2015).

Psychological therapies face different barriers. Cognitive behavioral therapy and CBT-I are effective, but access is constrained by workforce capacity, referral pathways, cost, travel, and engagement (Gould et al., 2012; Koffel et al., 2018; Elshaikh et al., 2023). Digital delivery may extend reach for some people, yet technological access, literacy, privacy concerns, and reliance on carers can exclude others (Pywell et al., 2020; Lu et al., 2022).

Aerobic and resistance exercises can improve health but may be difficult for older adults experiencing pain, fatigue, fear of injury, low confidence, transport barriers, or limited facilities (Franco et al., 2015). Falls-prevention programmes may also be rejected when they appear stigmatising or misaligned with personal goals (Yardley et al., 2006). These constraints do not diminish established treatments; they explain the need for adaptable adjuncts.

#### 3.1.4 Why scalable low-intensity interventions are needed

Low-threshold activity is relevant because physical activity is associated with a lower risk of depression even below conventional targets (Pearce et al., 2022). Irish longitudinal data linked sub-recommended activity to fewer depressive symptoms and lower odds of major depression (Laird et al., 2023), and another population cohort found lower depression risk across activity intensities and frequencies (Lima et al., 2024). These observational findings support feasibility and prevention hypotheses but do not establish treatment efficacy.

Low-intensity mind–body exercise may combine movement with breathing, attentional control, postural stability, balance confidence, motor learning, and group participation (Larkey et al., 2009; Wayne and Kaptchuk, 2008a; Zaccaro et al., 2018; Li et al., 2018; Cruwys et al., 2013). Network meta-analysis suggests possible anxiety and depression benefits, although interventions and comparators are heterogeneous (Dong et al., 2024). For chronic insomnia, direct Tai Chi evidence is stronger: A randomised trial found inferior immediate outcomes to CBT-I but non-inferior longer-term outcomes (Siu et al., 2025).

Group delivery may also address inactivity and unmet social needs. Social prescribing links people to community resources, but evidence for older adults remains heterogeneous and does not prove that any particular activity reduces loneliness (Bickerdike et al., 2017; Percival et al., 2022; Cooper et al., 2022). Tai Chi is nevertheless compatible with this model because it requires little equipment, can be adapted, and can be delivered in community, clinical, residential, or hybrid settings (Wayne and Kaptchuk, 2008a; Glasgow et al., 1999). Its rationale is therefore multicomponent and adjunctive, not curative.

### 3.2 Tai Chi as a low-intensity mind–body exercise intervention

#### 3.2.1 Defining low-intensity mind–body exercise

Low-intensity mind–body exercise combines mild-to-moderate movement with breathing, focused attention, postural control, body awareness, relaxation, and purposeful self-regulation (Larkey et al., 2009; Jahnke et al., 2010). The broader category includes Qigong, gentle yoga, and meditative movement (Larkey et al., 2009; Cramer et al., 2013). Evidence from these related practices can inform hypotheses, but it should not be presented as direct evidence for Tai Chi.

Tai Chi is a complex intervention with physical, cognitive, emotional, social, and cultural components (Wayne and Kaptchuk, 2008a). Trials must therefore specify style, sequence, dose, progression, instructor qualifications, home practice, comparator, and context (Wayne and Kaptchuk, 2008b). These features may influence feasibility and adherence as much as physiological intensity, particularly in frail or cognitively vulnerable populations.

Magnetic resonance imaging (MRI) reviews of heterogeneous mind–body practices report possible structural and functional differences in networks involved in cognitive control, interoception, emotion regulation, and memory (Zhang et al., 2021; Han et al., 2023). Because these reviews combine interventions and mostly small studies, they provide indirect, hypothesis-generating support for Tai Chi rather than evidence of a Tai Chi-specific neural mechanism.

#### 3.2.2 Deconstructing Tai Chi into core therapeutic components

A component model separates slow rhythmic movement, weight transfer, postural control, breathing, focused attention, motor sequencing, relaxation, and group practice (Larkey et al., 2009; Wayne and Kaptchuk, 2008a; Lan et al., 2013). This model improves replication but also exposes a causal challenge: Any observed effect may reflect exercise, attention, expectancy, instructor contact, social interaction, or their combination (Wayne and Kaptchuk, 2008b; Zaccaro et al., 2018).

Population-use and clinical reviews show that Tai Chi and Qigong already function as self-management practices for wellness, stress, musculoskeletal problems, and chronic symptoms (Jahnke et al., 2010; Birdee et al., 2009; Klein and Picard, 2017). Those sources describe acceptability and broad plausibility; they do not establish efficacy for a specific psychiatric diagnosis.

Tai Chi should therefore be reported as a behavioral package rather than a black box. Candidate components include neuromotor training, breath-linked autonomic regulation, attentional control, embodied self-efficacy, behavioral activation, and social participation (Wayne and Kaptchuk, 2008a; Zaccaro et al., 2018; Li et al., 2018; Cruwys et al., 2013; Bandura, 1977; Dimidjian et al., 2006). Component and mediation analyses are needed to determine which, if any, cause clinical change.

##### 3.2.2.1 Slow rhythmic movement, balance control, and postural stability

Tai Chi trains slow weight transfer, lower-limb coordination, postural stability, and movement confidence. In inactive adults aged at least 70 years, a 6-month randomised trial reduced falls, fall risk, and fear of falling and improved balance and performance (Li et al., 2005). In Parkinson’s disease, Tai Chi improved postural stability and function relative to resistance training or stretching (Li et al., 2012). These are direct Tai Chi findings, but their relevance to depression or anxiety is indirect because mental health was not the primary endpoint.

A tailored Tai Ji Quan programme also reduced falls more than stretching and multimodal exercise in older adults at high risk (Li et al., 2018). Meta-analyses report improvements in balance and flexibility, with effects varying by protocol, dose, and participant risk (Huang and Liu, 2015; Logghe et al., 2010; Chen et al., 2023). Better balance may reduce fear and avoidance, but this psychological pathway requires direct testing.

Accordingly, balance and function are established intervention targets; downstream effects on mood, participation, or self-efficacy remain plausible rather than proven.

##### 3.2.2.2 Breath regulation, attentional control, and body awareness

Slow breathing, attentional focus, interoceptive awareness, and relaxation are plausible self-regulatory components. Reviews of slow breathing, respiratory vagal stimulation, mindfulness, interoception, and heart rate variability biofeedback provide indirect models for autonomic regulation and emotional regulation (Zaccaro et al., 2018; Gerritsen and Band, 2018; Hölzel et al., 2011; Tang et al., 2015; Mehling et al., 2012; Lehrer and Gevirtz, 2014). Tai Chi is not equivalent to any of these interventions. One small Tai Chi trial in older adults with depressive symptoms reported changes in depression and heart rate variability (Liu et al., 2018), but it did not establish mediation. Autonomic regulation should therefore be tested prospectively rather than assumed.

##### 3.2.2.3 Group practice, social participation, and behavioral activation

Group Tai Chi may provide routine, mastery, peer contact, and low-stigma participation. Broader evidence links group membership and active social participation to depression recovery and lower isolation (Cruwys et al., 2013; Cattan et al., 2005; Dickens et al., 2011), while late-life depression models and behavioral activation support re-engagement with rewarding activities (Dimidjian et al., 2006; Fiske et al., 2009; Alexopoulos, 2005; Cuijpers et al., 2007). Loneliness, isolation, and weak social relationships are also associated with poorer mental and physical outcomes (Holt-Lunstad et al., 2010, 2015; Santini et al., 2020). These sources support a social and behavioral hypothesis, but—apart from the small hybrid trial discussed below—they are not Tai Chi-specific evidence.

#### 3.2.3 Reframing Tai Chi from traditional practice to standardised behavioral intervention

For research and implementation, Tai Chi must be specified as a reproducible, theory-informed complex intervention without erasing its cultural origin. The Medical Research Council (MRC) framework requires attention to components, mechanisms, context, acceptability, fidelity, scalability, equity, and sustainability (Skivington et al., 2021).

The Template for Intervention Description and Replication (TIDieR) checklist should be used to report materials, procedures, provider expertise, delivery, doses, tailoring, modifications, and fidelity (Hoffmann et al., 2014). The Consolidated Standards of Reporting Trials (CONSORT) extension for non-pharmacological treatments addresses comparator credibility, provider effects, co-interventions, adherence, and the practical limits of blinding (Boutron et al., 2017). Treatment delivery, receipt, and enactment should be assessed explicitly (Bellg et al., 2004); trial reports and protocols should also follow CONSORT and Standard Protocol Items: Recommendations for Interventional Trials (SPIRIT), respectively (Schulz et al., 2010; Chan et al., 2013).

#### 3.2.4 Suitability for older adults with heterogeneous physical and cognitive capacity

Adaptability makes Tai Chi potentially suitable for heterogeneous older populations. Pain, fatigue, fear of injury, low confidence, transport barriers, and unsuitable facilities commonly restrict physical activity (Franco et al., 2015), while medicalised falls-prevention programmes may be resisted when they threaten identity or autonomy (Yardley et al., 2006). A gentle, culturally or recreationally framed programme may reduce some of these barriers.

Community evidence supports functional feasibility. In 132 healthy older adults, 6 months of Tai Chi or Western exercise improved selected physical and mental-function measures compared with attention control (Taylor-Piliae et al., 2010). Mind–body exercise meta-analyses report possible cognitive benefits (Wu et al., 2019), and Tai Chi trials show balance benefits in Parkinson’s disease and high-fall-risk populations (Li et al., 2012, 2018). These studies are relevant to delivery and function but should not be reclassified as direct evidence for treating mental illness.

Low intensity does not mean universally appropriate. Frailty, unstable cardiovascular disease, severe balance or vestibular disorders, advanced dementia, uncontrolled pain, and high falls risk may require screening, seated or supported adaptations, closer supervision, and environmental modification. Scalability depends on matching movement complexity, progression, group size, and instructor skill to participant capacity.

### 3.3 Evidence across interconnected mental-health outcomes

#### 3.3.1 Depression

Depression is the most studied mental-health outcomes in Tai Chi research, but diagnostic and methodological heterogeneity limits interpretation. Evidence must distinguish major depressive disorder from elevated symptom scores, distress in chronic disease, and general mood outcomes (Wang et al., 2014; Zeng et al., 2023; Zhu et al., 2024). That distinction determines whether Tai Chi is considered adjunctive treatment, prevention, or health promotion (Noetel et al., 2024).

##### 3.3.1.1 Clinical depression versus depressive symptoms

The most defensible conclusion is that Tai Chi may reduce depressive symptoms, particularly in middle-aged and older adults. Reviews report favourable effects but also small samples, passive controls, heterogeneous protocols, and limited diagnostic confirmation (Wang et al., 2014; Zeng et al., 2023; Zhu et al., 2024; Chi et al., 2013; Sani et al., 2023). Dose–response signals have been reported, yet styles, comparators, and outcome variations preclude a single prescription (Zeng et al., 2023; Zhu et al., 2024).

Screening measures such as the Geriatric Depression Scale, the Hamilton Depression Rating Scale, and the Patient Health Questionnaire quantify symptoms but do not substitute for a structured diagnostic interview (Chi et al., 2013; Kroenke et al., 2001; Yesavage et al., 1982). Symptom improvement in community or chronic-disease samples therefore cannot establish Tai Chi as monotherapy for major depression. Broader exercise evidence supports exercise as treatment and prevention, but effects vary by modality, intensity, supervision, and comparator (Noetel et al., 2024; Pearce et al., 2022).

##### 3.3.1.2 Adjunctive role rather than replacement therapy

Direct adjunctive trials provide a balanced signal. In older adults with major depression who had responded incompletely to escitalopram, Tai Chi Chih and medication outperformed health education and medication for depressive symptoms and several secondary outcomes (Lavretsky et al., 2011). In a later, larger trial, both Tai Chi Chih and health education improved mood; Tai Chi Chih improved general health but was not superior for mood or cognition (Lavretsky et al., 2022). Exploratory neuroimaging findings from this programme suggest possible connectivity changes but do not establish a mechanism (Kilpatrick et al., 2022).

Smaller studies suggest acceptability, symptom improvement, and possible autonomic change (Liu et al., 2018; Yeung et al., 2012; Kong et al., 2019), but their designs do not support replacement of antidepressants, psychotherapy, collaborative care, or crisis intervention. Tai Chi is best positioned as an adjunct for selected patients, with standard assessment and escalation for severe depression, suicidality, psychosis, bipolar disorder, or marked functional impairment.

#### 3.3.2 Anxiety

Late-life anxiety is often somatic and intertwined with insomnia, pain, chronic disease, fear of falling, and cognitive concern (Wolitzky-Taylor et al., 2010; Lenze and Wetherell, 2011; Vink et al., 2008). Meta-analyses suggest possible reductions in anxiety symptoms with Tai Chi or broader traditional mind–body exercise, but studies are heterogeneous, rarely require a diagnosed anxiety disorder, and often use weak comparators (Kuang et al., 2024; Dong et al., 2024; Wang et al., 2014; Feng et al., 2025; Zhang et al., 2019). Indirect evidence on breathing and attention supports plausibility, not Tai Chi-specific efficacy (Zaccaro et al., 2018).

The strongest anxiety-related rationale may concern physiological arousal, movement avoidance, and fear of falling. Tai Chi and therapeutic Tai Ji Quan improve balance and reduce falls or fear of falling in selected older populations (Li et al., 2005, 2018; Logghe et al., 2010). These outcomes may support confidence and participation, but they do not demonstrate treatment of generalised anxiety disorder, panic disorder, or phobic disorder. Disorder-specific trials with diagnostic assessment and active controls are needed.

#### 3.3.3 Insomnia and sleep disturbance

Sleep is the clearest clinical entry point for Tai Chi in late-life mental health. Insomnia commonly overlaps with depression, anxiety, pain, frailty, medication burden, and cognitive vulnerability (Baglioni et al., 2011; Patel et al., 2018; Yaffe et al., 2014). Unlike much of the mood literature, sleep research includes large randomised trials with objective and subjective measures (Siu et al., 2021, 2025).

##### 3.3.3.1 Tai Chi as a behavioral sleep intervention

In 320 adults aged at least 60 years with chronic insomnia, 12 weeks of Tai Chi and conventional exercises improved actigraphy-measured sleep relative to no intervention, and gains were maintained over 24 months; Tai Chi was not superior to conventional exercise, and absolute changes were modest (Siu et al., 2021). In 200 adults aged at least 50 years, Tai Chi was inferior to CBT-I immediately after a 3-month programme but met the prespecified non-inferiority criterion at 15 months (Siu et al., 2025). Meta-analyses report improved self-rated sleep, although many studies rely on subjective outcomes and heterogeneous protocols (Du et al., 2015; Raman et al., 2013; Lyu et al., 2024). The review of exercise in older women reaches a similarly favourable but heterogeneous conclusion (Khaleghi and Ahmadi, 2024).

CBT-I remains first-line treatment for chronic insomnia in clinical guidelines (Edinger et al., 2021; Qaseem et al., 2016; Riemann et al., 2017). Tai Chi may be considered an adjunct, a lower-intensity option when CBT-I is inaccessible or declined, or a maintenance component within stepped care. Long-term non-inferiority should not be interpreted as immediate equivalence or as a reason to withhold CBT-I.

##### 3.3.3.2 Sleep as a mediator of mood and cognitive outcomes

Sleep may connect Tai Chi with mood and cognition, but mediation has not been established. Insomnia predicts depression (Li et al., 2016; Baglioni et al., 2011), sleep and depression may be bidirectional (Bao et al., 2017), and sleep disturbance is associated with dementia risk (Shi et al., 2018; Yaffe et al., 2014). Tai Chi could influence sleep through daytime activity, reduced arousal, breathing, routine, pain relief, or reduced rumination (Siu et al., 2021, 2025). Future trials should test whether changes in sleep efficiency, circadian regularity, insomnia severity, or hypnotic use mediate subsequent mood or cognitive outcomes.

#### 3.3.4 Loneliness and social isolation

Loneliness and social isolation are important late-life targets, but direct Tai Chi evidence is substantially weaker than evidence for sleep, depressive symptoms, or balance (Holt-Lunstad et al., 2015; Santini et al., 2020). Group Tai Chi may be a practical social-prescribing activity because it combines movement, routine, and contact; that implementation rationale is not proof of an anti-loneliness effect (Bickerdike et al., 2017; Percival et al., 2022; Cattan et al., 2005; Dickens et al., 2011).

##### 3.3.4.1 Group-based Tai Chi as a social-prescribing vehicle

Social prescribing links patients to non-clinical community resources. Reviews in older adults and mental-health settings report possible benefit but substantial heterogeneity and weak causal designs (Bickerdike et al., 2017; Percival et al., 2022; Cooper et al., 2022). Realist work suggests that trusted referral, link-worker support, acceptable activities, and sustained engagement determine outcomes (Husk et al., 2020; Elliott et al., 2022). This contextual evidence informs delivery of Tai Chi but is not evidence that Tai Chi itself reduces loneliness.

Tai Chi is compatible with community referral because it can be offered through primary care, rehabilitation, senior centres, public health services, or residential care. Broader reviews suggest that actively participatory, theory-based group interventions are more promising than passive information for social isolation (Cattan et al., 2005; Dickens et al., 2011), and group identification may support depression recovery (Cruwys et al., 2013). Programmes must nevertheless address referral quality, accessibility, instructor competence, cultural fit, and retention.

##### 3.3.4.2 The gap between plausible mechanism and direct evidence

Loneliness and social isolation are associated with depression, anxiety, mortality, cognitive decline, and dementia risk (Penninkilampi et al., 2018; Holt-Lunstad et al., 2010, 2015; Santini et al., 2020). A meta-analysis of more than 600,000 participants further associated loneliness with incident dementia (Luchetti et al., 2024). These observational associations may reflect confounding, reverse causation, or prodromal disease and therefore identify risk, not a Tai Chi treatment effect.

Direct evidence remains sparse. A 48-participant pilot trial in socially isolated ‘hidden elders’ reported possible improvements in loneliness and social support after peer-assisted Tai Chi Qigong (Chan et al., 2017). Because the programme combined Qigong-style movement with volunteer contact, its effect cannot be attributed to Tai Chi alone. Future trials should prespecify validated measures of subjective loneliness and objective isolation (National Academies of Sciences, Engineering, and Medicine, 2020; Russell, 1996; Lubben et al., 2006) and use active social comparators to separate movement from attention, expectancy, and group contact (Hoffmann et al., 2014; Boutron et al., 2017).

#### 3.3.5 Cognitive decline and dementia risk

Cognition is a promising but easily overstated domain. Tai Chi combines movement, balance, sequencing, attention, working memory, and dual-task coordination (Zhang et al., 2021; Han et al., 2023; Wu et al., 2019). Improvement on a cognitive test, however, is not evidence that Tai Chi prevents dementia (Livingston et al., 2024; Chen et al., 2024; Shao et al., 2024).

##### 3.3.5.1 Subjective cognitive decline and mild cognitive impairment

Meta-analyses suggest possible improvements in global cognition and selected domains among older adults with MCI while repeatedly noting small studies, heterogeneous protocols, and uncertain clinical importance (Chen et al., 2024; Shao et al., 2024; Rampengan et al., 2024). Evidence in early dementia is limited and short-term (Lim et al., 2019), and prospective evidence in cognitively healthy adults remains methodologically weak (Zheng et al., 2015). A 2023 randomised trial of 318 older adults with MCI or memory concerns found that cognitively enhanced Tai Ji Quan improved global cognition and dual-task walking more than standard Tai Ji Quan or stretching at 24 weeks, with some maintenance at 48 weeks (Li et al., 2023). The trial had no non-exercise control and a predominantly high-functioning, educated, White sample; it did not assess dementia incidence.

Executive function, attention, processing speed, visuospatial ability, memory, and dual-task control are plausible targets because practice requires sequence learning, weight transfer, limb coordination, postural monitoring, and sustained attention. Broader mind–body evidence supports a cognitive–motor hypothesis (Wu et al., 2019; Park et al., 2023), but mechanistic studies in MCI or early dementia remain sparse (Jasim et al., 2023). Tai Chi is therefore a candidate cognitive–motor intervention, not an established cognitive rehabilitation or disease-modifying therapy.

##### 3.3.5.2 Why current evidence does not justify dementia-prevention claims

Dementia prevention is multidomain and includes physical inactivity, depression, social isolation, hearing loss, hypertension, diabetes, smoking history, obesity, air pollution, traumatic brain injury, excessive alcohol consumption, low education level, visual loss, and high low-density lipoprotein (LDL) cholesterol level (Livingston et al., 2024). Tai Chi may influence activity, sleep, mood, balance, and participation, but this does not demonstrate reduced dementia incidence. Observational exercise evidence is vulnerable to confounding and reverse causation (Kivimäki et al., 2019), and exercise trials in dementia or MCI remain heterogeneous and inconclusive (Jasim et al., 2023; Forbes et al., 2015; Gates et al., 2013).

Tai Chi may contribute to a multidomain healthy brain strategy, but prevention claims require adequately powered, long-term trials assessing incident dementia, conversion from MCI, or validated cognitive trajectories (Livingston et al., 2024; Ngandu et al., 2015). Current studies are generally too small, brief, and reliant on intermediate tests for such claims. Table 1 summarises the populations, protocols, comparators, outcomes, follow-ups, findings, and limitations of 10 key trials.

### 3.4 Mechanisms, dose, delivery, and implementation

#### 3.4.1 Candidate mechanisms underlying transdiagnostic effects

Tai Chi is unlikely to affect late-life mental health through one pathway. Candidate pathways include autonomic regulation, sleep and circadian stability, cognitive–motor learning, self-efficacy, balance confidence, behavioral activation, and social participation (Wayne and Kaptchuk, 2008a; Skivington et al., 2021). The pathways may interact, but most evidence is indirect, and few trials have tested mediation. Figure 1 is therefore a hypothesis-generating model, not a causal diagram (Baglioni et al., 2011; Zaccaro et al., 2018; Li et al., 2018; Cruwys et al., 2013; Wu et al., 2019).

**Figure 1:**
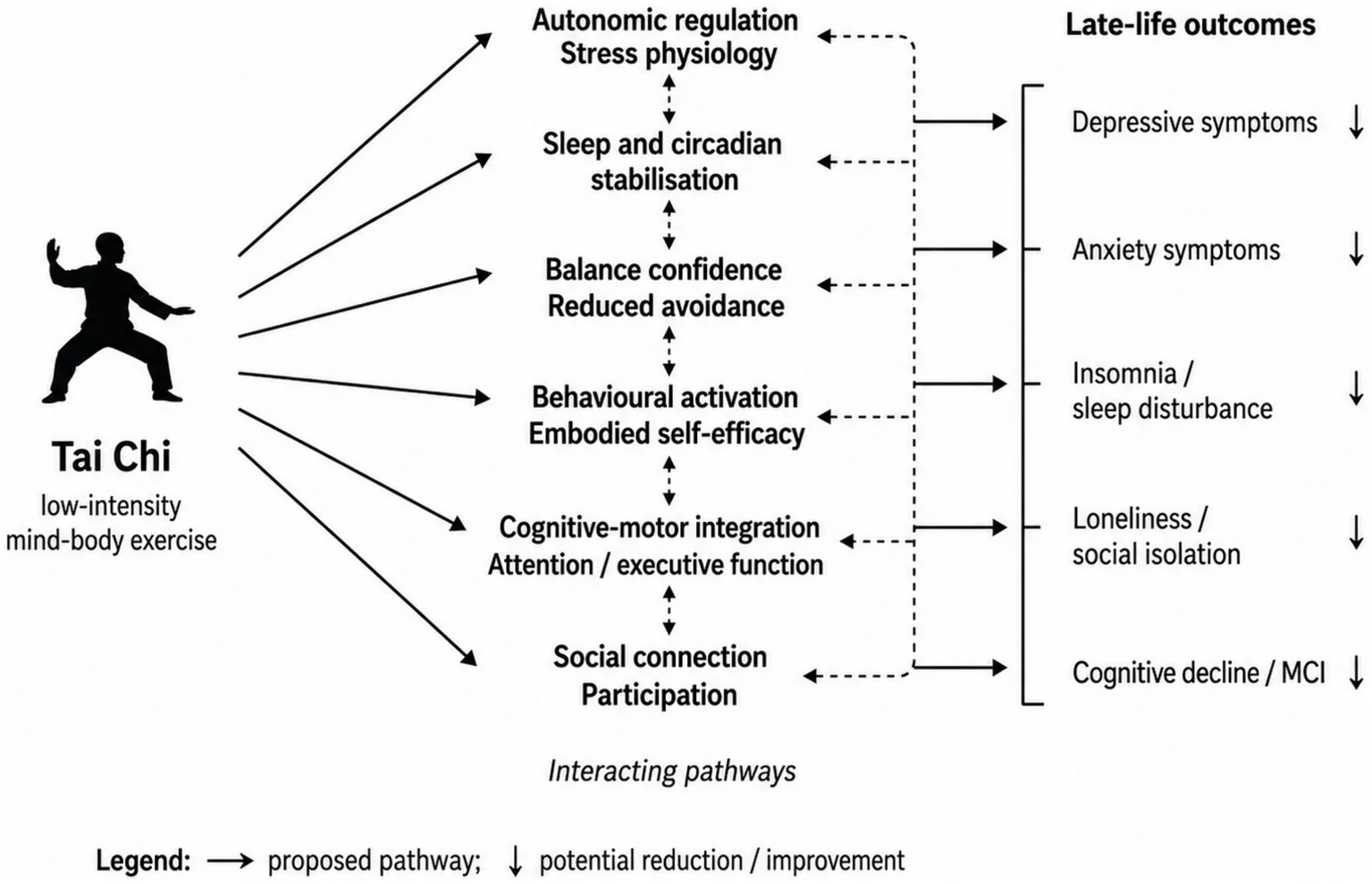
Proposed transdiagnostic pathways linking Tai Chi to mental-health and cognitive outcomes in older adults. Tai Chi is conceptualised as a low-intensity, multicomponent mind–body intervention. Candidate pathways include autonomic and stress regulation, sleep and circadian stabilisation, balance confidence and reduced avoidance, behavioral activation and self-efficacy, cognitive–motor integration, and social participation. The majority of supporting evidence is indirect, associative, or exploratory. The model does not establish mediation, psychiatric efficacy, or dementia prevention.

##### 3.4.1.1 Autonomic regulation and stress physiology

Slow movement, breathing, attention, and relaxation make autonomic regulation a plausible pathway. Slow-breathing and heart rate variability biofeedback studies provide indirect models involving cardiorespiratory coupling, baroreflex activity, and vagal afferent signalling (Zaccaro et al., 2018; Gerritsen and Band, 2018; Lehrer and Gevirtz, 2014). They are not Tai Chi trials and cannot establish a Tai Chi-specific mechanism.

The neurovisceral integration model links heart rate variability to prefrontal and autonomic regulation (Thayer and Lane, 2009). One small Tai Chi trial in older adults with depression reported changes in heart rate variability and symptoms (Liu et al., 2018), while broader mind–body research implicates autonomic and inflammatory processes (Pascoe et al., 2017). Because mediation, repeated physiology, and adequate active controls are largely absent, autonomic change remains a testable hypothesis rather than an established mediator.

##### 3.4.1.2 Sleep and circadian stabilisation

Sleep is another candidate pathway. Insomnia predicts depression; sleep and mood may influence each other, and sleep disturbance is associated with cognitive impairment and dementia risk (Li et al., 2016; Shi et al., 2018; Baglioni et al., 2011; Yaffe et al., 2014; Bao et al., 2017). Sleep also interacts with inflammatory and emotional regulation (Irwin, 2015). Direct trials show that Tai Chi can improve sleep relative to no intervention, but it performed similarly to conventional exercise in one trial and was inferior to CBT-I immediately after treatment in another (Siu et al., 2021, 2025). Mediation analyses are needed before sleep improvement can explain downstream mood or cognitive change.

##### 3.4.1.3 Cognitive–motor integration and neuroplasticity

Tai Chi requires sequence learning, weight transfer, bilateral coordination, postural monitoring, speed control, and sustained attention, making cognitive–motor training plausible (Wayne and Kaptchuk, 2008a; Lan et al., 2013; Wu et al., 2019). Reviews of Tai Chi, Qigong, and other mind–body practices report possible cognitive or neuroimaging effects (Zhang et al., 2021; Han et al., 2023; Park et al., 2023), and one MRI study found differences relative to aerobic exercise (Cui et al., 2019). Because interventions, samples, and imaging methods vary and studies are small, these findings remain preliminary; the MCI literature likewise lacks robust mechanistic mediation (Jasim et al., 2023).

##### 3.4.1.4 Embodied self-efficacy and social connection

Mastery of safe movement may enhance self-efficacy, balance confidence, and willingness to participate (Bandura, 1977). Falls trials show improved balance and function (Li et al., 2005, 2012, 2018), but psychological mediation was not established. Group practice may also provide routine, peer contact, and behavioral activation; broader social-group and behavioral-activation evidence supports this possibility (Santini et al., 2020; Cruwys et al., 2013; Dimidjian et al., 2006; Holt-Lunstad et al., 2010). Active social and movement comparators are needed to isolate any Tai Chi-specific effect.

#### 3.4.2 Dose, fidelity, and intervention standardisation

Research should replace generic efficacy claims with reproducible prescriptions. Without a specified dose, instructor model, adherence measure, and credible comparator, inconsistent findings cannot be attributed to the intervention rather than implementation failure or study design.

##### 3.4.2.1 Frequency, duration, intensity, and programme length

Dose should include session frequency and duration, programme length, movement intensity, progression, home practice, and maintenance. TIDieR and fidelity guidance require reporting what was planned, delivered, received, and enacted (Hoffmann et al., 2014; Bellg et al., 2004). Existing programmes vary widely (Zeng et al., 2023; Lyu et al., 2024), and Tai Chi methodology reviews call for explicit style, instructor background, class size, and home practice (Lan et al., 2013; Wayne and Kaptchuk, 2008b).

Dose may matter for depressive symptoms, sleep, and falls, but current dose–response findings are exploratory (Zeng et al., 2023; Zhu et al., 2024; Lyu et al., 2024). Sustained programmes have produced long-term sleep and fall outcomes (Li et al., 2018; Siu et al., 2021). Future trials should report intended, attended, and home-practice doses; achieved intensity; progression; adherence; and prespecified dose–response analyses.

##### 3.4.2.2 Tai Chi style, instructor training, and protocol reproducibility

Tai Chi is not homogeneous. Yang, Chen, Sun, simplified 24-form, Tai Chi Chih, and therapeutic Tai Ji Quan differ in stance, tempo, balance challenge, breathing instruction, sequence complexity, and suitability for frailty. Indirect comparisons suggest that form may influence mood outcomes, but the network evidence is unknown (Kuang et al., 2024). The multicomponent theory reinforces the need to define content, teacher expertise, progression, context, and comparator (Wayne and Kaptchuk, 2008a,b).

Standardisation requires complete TIDieR reporting of materials, procedures, providers, delivery, doses, tailoring, modifications, and fidelity (Hoffmann et al., 2014). CONSORT guidance is needed for provider expertise, adherence, co-interventions, comparator credibility, and blinding (Boutron et al., 2017). The National Institutes of Health (NIH) Behavior Change Consortium framework further distinguishes study design, provider training, delivery, receipt, and enactment (Bellg et al., 2004). Community exercise work using an adapted TIDieR framework illustrates how protocol audit can support implementation fidelity (Bird et al., 2020).

#### 3.4.3 Delivery models for ageing societies

Public health value depends on safe, affordable, and equitable delivery under real-world conditions. Reach, adoption, implementation, maintenance, context, and sustainability are also important in addition to efficacy (Glasgow et al., 1999; Skivington et al., 2021). Candidate settings include community services, primary care, residential care, rehabilitation, and hybrid delivery. Social prescribing and digital delivery may expand access, but their effectiveness and equity cannot be assumed (Pywell et al., 2020; Lu et al., 2022; Bickerdike et al., 2017; Percival et al., 2022).

##### 3.4.3.1 Community-based group programmes

Community groups align with Tai Chi’s usual format and may support routine, peer interaction, modelling, and low-stigma engagement. They may also reduce barriers such as low confidence or unsuitable facilities (Franco et al., 2015). Acceptance nevertheless depends on identity, goals, cultural fit, transport, cost, and whether the programme is framed as supportive rather than stigmatising (Yardley et al., 2006).

Falls trials support community feasibility: Tai Chi and therapeutic Tai Ji Quan reduced falls and improved functional outcomes in inactive or high-risk older adults (Li et al., 2005, 2018, 2019a). A cost-effectiveness analysis also found therapeutic Tai Ji Quan economically attractive for fall prevention in a defined high-risk population (Li et al., 2019b). These findings concern falls—not mental-health effectiveness—but function and safety are prerequisites for sustained participation.

##### 3.4.3.2 Primary care referral and social prescribing

Primary care may refer older adults with mild symptoms, inactivity, sleep disturbance, social withdrawal, pain, or multimorbidity to community programmes. Social-prescribing reviews report promising but heterogeneous evidence (Bickerdike et al., 2017; Percival et al., 2022; Cooper et al., 2022), and realist reviews show that trusted referral, link-worker support, activity fit, and sustained engagement influence outcomes (Husk et al., 2020; Elliott et al., 2022).

Accordingly, Tai Chi referral should be evaluated as a complex pathway rather than a simple prescription. Process evaluation should capture who is referred, uptake, reach, fidelity, context, retention, outcomes, and unintended effects (Skivington et al., 2021).

Referral pathways require clinical risk assessments, qualified instructors, feedback to referring services, accessibility planning, and escalation routes for suicidality, psychosis, severe depression, unstable medical illness, or advanced cognitive impairment. Intervention content, provider expertise, dose, tailoring, adherence, and fidelity should be specified (Skivington et al., 2021; Hoffmann et al., 2014; Bellg et al., 2004).

##### 3.4.3.3 Residential aged care, rehabilitation, and digital delivery

Residential aged-care and rehabilitation settings are important but under-studied. Frailty, cognitive impairment, polypharmacy, staffing constraints, and falls risk may require simplified, seated, supported, or individually progressed protocols. Mixed exercise evidence in dementia cautions against assuming benefit (Forbes et al., 2015), while multidomain dementia guidance supports non-pharmacological activity only as one component of care (Livingston et al., 2024).

Rehabilitation evidence is strongest for function: Tai Chi improved postural stability in Parkinson’s disease, and therapeutic Tai Ji Quan reduced falls in high-risk older adults (Li et al., 2012, 2018, 2019a). Sleep trials show that a structured programme can be sustained (Siu et al., 2021). Translation to rehabilitation-related mental-health outcomes still requires direct evaluation.

Digital and hybrid delivery may help people with transport, rural-access, caregiving, or mobility barriers. Small videoconference studies demonstrate feasibility in selected older adults, including those with MCI (Li et al., 2021, 2022), but do not establish population-wide scalability. Remote instruction can limit posture correction and detection of fatigue or falls risk (Montero-Odasso et al., 2022). Wearable monitoring remains developmental (Corniani et al., 2025). Hybrid models should test an initial in-person assessment followed by remote maintenance while measuring safety, fidelity, digital literacy, and equity (Pywell et al., 2020; Lu et al., 2022; Hoffmann et al., 2014).

#### 3.4.4 Safety, accessibility, and health equity

Tai Chi is low impact but not risk-free. Frailty, orthostatic hypotension, vestibular disease, osteoarthritis, osteoporosis, neuropathy, visual impairment, unstable cardiovascular disease, advanced dementia, and medication-related falls risk may require adaptation or supervision. World Health Organization (WHO) guidance recommends multicomponent activity adapted to older adults’ abilities (Bull et al., 2020), and international falls guidance supports individual risk assessment and tailored balance exercise (Montero-Odasso et al., 2022). Sedatives, anticholinergics, antidepressants, antipsychotics, and polypharmacy may further affect safety (American Geriatrics Society Beers Criteria Update Expert Panel, 2023).

Access is influenced by pain, fatigue, fear, transport, cost, confidence, cultural relevance, prior exercise experience, and programme fit (Franco et al., 2015; Yardley et al., 2006). Tai Chi may be acceptable to some populations, but preference should not be generalised across cultures, languages, disability levels, socioeconomic groups, or health systems. A healthy-ageing approach should prioritise function and participation (Beard et al., 2016).

Equity should be designed and measured. Social position affects access to safe venues, transport, trained instructors, digital tools, and sustained programmes (Marmot, 2005; O’Neill et al., 2014; Welch et al., 2012). Low equipment cost is helpful but insufficient. Programmes should address language, disability adaptation, transport, digital exclusion, instructor diversity, subsidised access, and links to health and social care (Pywell et al., 2020; Lu et al., 2022). Reach among isolated, frail, and underserved adults is a substantive outcome, not an implementation footnote.

### 3.5 Future research agenda and public health implications

#### 3.5.1 Key limitations of the current evidence base

The evidence base has progressed beyond anecdote, but it remains insufficient for broad disease-treatment or dementia-prevention claims. Recurrent limitations include small single-centre trials, heterogeneous protocols, passive comparators, inconsistent outcomes, limited follow-ups, incomplete harm reporting, and little economic evaluation. These problems affect both internal validity and real-world scalability.

##### 3.5.1.1 Small samples, heterogeneous interventions, and weak comparators

Many trials are underpowered and vary in style, doses, instructor expertise, home practice, participant frailty, baseline severity, and comparators (Wayne and Kaptchuk, 2008a,b). The MRC complex-intervention framework requires evaluation of mechanisms, context, implementation, acceptability, equity, and scalability as well as outcomes (Skivington et al., 2021).

Wait-list, usual-care, or minimal-education controls do not adequately separate Tai Chi from attention, expectancy, social contact, instructor enthusiasm, gentle movement, or behavioral activation. Active comparators and complete reporting under CONSORT and TIDieR are therefore essential (Hoffmann et al., 2014; Boutron et al., 2017). Meta-analyses across depression, anxiety, and cognition repeatedly identify small samples and heterogeneity as major constraints (Kuang et al., 2024; Chen et al., 2024; Wang et al., 2014).

##### 3.5.1.2 Inconsistent outcome measures and insufficient long-term follow-ups

Outcome heterogeneity also impedes synthesis. Trials variously assess symptoms, diagnoses, sleep, cognition, balance, falls, function, quality of life, or wellbeing. Core outcome sets and validated, responsive, interpretable instruments would reduce selective reporting and improve comparability (Williamson et al., 2017; Mokkink et al., 2018).

Depression, anxiety, insomnia, cognition, loneliness, and isolation should be measured directly with validated tools—for example, the Patient Health Questionnaire-9, the Generalized Anxiety Disorder-7, the Insomnia Severity Index, the Pittsburgh Sleep Quality Index, domain-specific cognitive tests, and validated loneliness or network measures (Kroenke et al., 2001; Spitzer et al., 2006; Bastien et al., 2001; Buysse et al., 1989; Nasreddine et al., 2005; Russell et al., 1980). A global wellbeing score cannot substitute for a prespecified clinical endpoint.

Follow-ups should distinguish immediate response, maintenance, relapse, sustained activity, medication change, function, cognitive trajectory, and health service use. Sleep trials illustrate why timing is important: Immediate and long-term comparisons can yield different conclusions (Siu et al., 2021, 2025).

##### 3.5.1.3 Poor reporting of adverse events and implementation costs

Harms should be prospectively defined, actively collected, analysed, and reported under the CONSORT harms extension (Ioannidis et al., 2004). Provider expertise, delivery, adherence, co-interventions, and adaptations are also needed to interpret safety (Boutron et al., 2017). In frail populations, falls-risk assessment and tailored progression should follow international guidance (Montero-Odasso et al., 2022).

Trials should record comorbidity, medication burden, mobility aids, pain, dizziness, baseline falls risk, and events during supervised and home practice. The Beers Criteria are relevant to sedative, anticholinergic, antipsychotic, and other medication-related risks (American Geriatrics Society Beers Criteria Update Expert Panel, 2023), stating that only no serious event occurred is inadequate unless surveillance methods and denominators are reported.

Economic analyses should capture instructor training, venues, referral, transport, digital infrastructure, screening, fidelity monitoring, and maintenance. The Consolidated Health Economic Evaluation Reporting Standards (CHEERS) 2022 statement and health-economic methods require an explicit perspective, time horizon, comparator, resource use, outcomes, and uncertainty (Husereau et al., 2022; Sanders et al., 2016). Falls-prevention evidence shows that Tai Ji Quan can be cost-effective in a specified high-risk group (Li et al., 2019b); equivalent evidence is absent for mental-health, sleep, loneliness, and cognitive outcomes.

#### 3.5.2 Priorities for next-generation trials

Next-generation studies should specify who the intervention is for, the protocol and dose, provider competence, comparator, mechanism, harms, cost, setting, and maintenance strategy. The aim is to answer clinically actionable questions rather than repeat an undifferentiated ‘Does Tai Chi work?’ comparison.

##### 3.5.2.1 Pragmatic multicentre randomised trials

Pragmatic multicentre trials should test diverse participants, instructors, settings, and health systems. The CONSORT pragmatic extension guides reporting of eligibility, setting, flexibility, adherence, and applicability (Zwarenstein et al., 2008). The Pragmatic Explanatory Continuum Indicator Summary 2 (PRECIS-2) tool and its predecessor can align eligibility, recruitment, setting, organisation, delivery, follow-up, outcomes, and analysis with the decision the trial is intended to inform (Loudon et al., 2015; Thorpe et al., 2009).

Process evaluation should accompany effectiveness studies because context, provider skill, fidelity, engagement, and organisational support may determine results. MRC guidance recommends evaluating implementation, mechanisms of impact, and context (Skivington et al., 2021; Moore et al., 2015), while pragmatic-trial methods emphasise broad eligibility and clinically meaningful outcomes (Ford and Norrie, 2016). Community centres, primary care, aged care, rehabilitation, and hybrid platforms should be represented.

##### 3.5.2.2 Active comparator designs and blinded outcome assessments

Comparators should match attention, expectancy, social contact, and physical activity where relevant. Options include stretching, health education, walking, balance or resistance training, CBT-I, mindfulness, dance, or social groups. Both experimental and comparator arms require equivalent TIDieR and CONSORT description (Hoffmann et al., 2014; Boutron et al., 2017).

Participants and instructors usually cannot be blinded, but allocation procedures, prespecified analyses, blinded outcome assessments, and blinded adjudication of objective outcomes remain possible. Lack of blinding can bias estimates, especially for subjective outcomes (Hróbjartsson et al., 2012; Savović et al., 2012). CONSORT and SPIRIT require transparent reporting and prospective specification of outcomes, comparator rationale, blinding, attrition, analyses, and harms (Schulz et al., 2010; Chan et al., 2013).

##### 3.5.2.3 Mechanistic biomarkers, objective sleep measures, and cost-effectiveness

Mechanism trials should measure candidate pathways rather than infer them from symptom change. Autonomic hypotheses require repeated heart rate variability, respiration, cardiovascular measures, and stress reactivity (Zaccaro et al., 2018; Lehrer and Gevirtz, 2014). Inflammatory hypotheses require prespecified biomarkers relevant to depression and ageing (Dowlati et al., 2010; Miller and Raison, 2016). Neuroplasticity hypotheses require validated cognitive tests, neuroimaging, or biomarkers such as brain-derived neurotrophic factor, with temporal and mediation analyses (Dinoff et al., 2016).

Sleep studies should pair validated self-report with actigraphy, sleep diaries, circadian measures, medication use, and extended follow-up (Siu et al., 2021; Ancoli-Israel et al., 2003; Smith et al., 2018). Economic evaluation should be embedded prospectively and reported under the CHEERS (Husereau et al., 2022). The fall-prevention cost-effectiveness study (Li et al., 2019b) demonstrates feasibility but cannot be extrapolated to depression, anxiety, insomnia, loneliness, or cognition.

#### 3.5.3 Clinical positioning of Tai Chi

Clinically, Tai Chi is an adjunct rather than a substitute for established treatment. It may help selected older adults with depressive symptoms, inactivity, sleep disturbance, chronic disease, or balance-related anxiety, but evidence is insufficient to replace medication, psychotherapy, collaborative care, or crisis intervention (Noetel et al., 2024; Kuang et al., 2024; Dong et al., 2024; Wang et al., 2014; Lavretsky et al., 2011).

For chronic insomnia, Tai Chi may be an adjunct, maintenance option, or lower-intensity alternative when CBT-I is inaccessible or declined; CBT-I remains first-line care (Edinger et al., 2021; Siu et al., 2025; Qaseem et al., 2016). For subjective cognitive decline or MCI, Tai Chi can form part of a multidomain activity and rehabilitation plan, but it should not be described as proven dementia prevention (Livingston et al., 2024; Chen et al., 2024; Li et al., 2023).

Referral should follow assessment of falls, frailty, cardiovascular stability, pain, cognition, medication burden, and psychiatric severity, with clear guidance on intensity, supervision, progression, and escalation. Tai Chi is most defensible for mild-to-moderate symptoms and functional vulnerability, not as a primary treatment for severe mental illness.

#### 3.5.4 Public health positioning of Tai Chi in healthy ageing systems

At the population level, Tai Chi fits healthy-ageing infrastructure better than a disease-specific therapy. World Health Organization (WHO) frameworks emphasise functional ability, intrinsic capacity, supportive environments, participation, and multicomponent physical activity (Beard et al., 2016; Bull et al., 2020). Tai Chi may address several of these domains, but multidomain dementia-risk guidance should not be reduced to one exercise (Livingston et al., 2024).

Programmes could be embedded in community services, primary-care referral, rehabilitation, residential care, falls prevention, and social prescribing. Reviews support careful pathway design but do not establish social-prescribing or Tai Chi-specific efficacy (Bickerdike et al., 2017; Percival et al., 2022; Husk et al., 2020). Equity requires trained instructors, risk screening, disability adaptation, transport, culturally and linguistically appropriate communication, affordable access, and links to health and social care (Marmot, 2005). Public health success should be judged by reach, sustained participation, safety, and clinically meaningful outcomes among isolated, frail, and underserved older adults.

## 4 Conclusion

Tai Chi is not a cure for mental illness or a proven dementia-prevention strategy. Its most credible role is as an adaptable, low-impact, socially deliverable adjunct for overlapping late-life vulnerabilities. Evidence is comparatively strongest for depressive symptoms, sleep disturbance, balance, and selected cognitive measures, while evidence for diagnosed psychiatric disorders, loneliness as a primary outcome, long-term cognitive trajectories, and dementia incidence remains limited. Proposed autonomic, sleep, cognitive–motor, self-efficacy, behavioral, and social pathways require direct mediation tests. Future trials should specify dose and fidelity, use credible active comparators and blinded assessment where feasible, follow participants long enough to assess maintenance, collect harms systematically, embed economic evaluation, and measure reach and equity. If these standards are met, Tai Chi may become a useful component of integrated mental-health and healthy-ageing systems; current evidence supports cautious implementation and evaluation, not overstatement.

## Appendix A. Reproducible PubMed search strategy

All searches were run in PubMed on 30 June 2026. The publication-date limit was applied to every query. Related-practice terms were included to identify mechanistic or implementation evidence and were not treated as Tai Chi-specific clinical evidence.

### Clinical-outcome search

((“Tai Chi”[Title/Abstract] OR “Tai Ji”[Title/Abstract] OR “Tai Ji Quan”[Title/Abstract] OR “Tai Chi Chuan”[Title/Abstract] OR Taiji[Title/Abstract] OR Taijiquan[Title/Abstract] OR Qigong[Title/Abstract] OR “meditative movement”[Title/Abstract] OR “mind–body exercise”[Title/Abstract]) AND (“Aged”[MeSH Terms] OR “Aging”[MeSH Terms] OR “older adult”[Title/Abstract] OR “older adults”[Title/Abstract] OR elderly[Title/Abstract] OR ageing[Title/Abstract] OR aging[Title/Abstract] OR geriatric[Title/Abstract] OR “late-life”[Title/Abstract]) AND (“Mental Health”[MeSH Terms] OR “Depression”[MeSH Terms] OR “Anxiety”[MeSH Terms] OR “Sleep Wake Disorders”[MeSH Terms] OR “Loneliness”[MeSH Terms] OR “Social Isolation”[MeSH Terms] OR “Cognitive Dysfunction”[MeSH Terms] OR “Dementia”[MeSH Terms] OR depression[Title/Abstract] OR depressive[Title/Abstract] OR anxiety[Title/Abstract] OR insomnia[Title/Abstract] OR “sleep disturbance”[Title/Abstract] OR “sleep quality”[Title/Abstract] OR loneliness[Title/Abstract] OR “social isolation”[Title/Abstract] OR “social participation”[Title/Abstract] OR cognition[Title/Abstract] OR “cognitive decline”[Title/Abstract] OR “mild cognitive impairment”[Title/Abstract] OR dementia[Title/Abstract])) AND (“1900/01/01”[Date - Publication]: “2026/06/30”[Date - Publication])

### Mechanism search

((“Tai Chi”[Title/Abstract] OR “Tai Ji Quan”[Title/Abstract] OR Taijiquan[Title/Abstract] OR Qigong[Title/Abstract] OR “mind–body exercise”[Title/Abstract]) AND (“older adult”[Title/Abstract] OR “older adults”[Title/Abstract] OR elderly[Title/Abstract] OR ageing[Title/Abstract] OR aging[Title/Abstract]) AND (“heart rate variability”[Title/Abstract] OR autonomic[Title/Abstract] OR inflammation[Title/Abstract] OR circadian[Title/Abstract] OR neuroplasticity[Title/Abstract] OR interoception[Title/Abstract] OR “brain-derived neurotrophic factor”[Title/Abstract] OR MRI[Title/Abstract])) AND (“1900/01/01”[Date - Publication]: “2026/06/30”[Date - Publication])

### Implementation, safety, and economic search

((“Tai Chi”[Title/Abstract] OR “Tai Ji Quan”[Title/Abstract] OR Taijiquan[Title/Abstract] OR Qigong[Title/Abstract] OR “mind–body exercise”[Title/Abstract]) AND (“older adult”[Title/Abstract] OR “older adults”[Title/Abstract] OR elderly[Title/Abstract] OR ageing[Title/Abstract] OR aging[Title/Abstract]) AND (implementation[Title/Abstract] OR fidelity[Title/Abstract] OR adherence[Title/Abstract] OR “dose–response”[Title/Abstract] OR safety[Title/Abstract] OR “adverse event”[Title/Abstract] OR “adverse events”[Title/Abstract] OR cost-effectiveness[Title/Abstract] OR “social prescribing”[Title/Abstract] OR “primary care”[Title/Abstract] OR digital[Title/Abstract] OR equity[Title/Abstract])) AND (“1900/01/01”[Date - Publication]: “2026/06/30”[Date - Publication])
